# When is it impractical to ask informed consent? A systematic review

**DOI:** 10.1177/17407745221103567

**Published:** 2022-07-01

**Authors:** Sara JM Laurijssen, Rieke van der Graaf, Wouter B van Dijk, Ewoud Schuit, Rolf HH Groenwold, Diederick E Grobbee, Martine C de Vries

**Affiliations:** 1 Department of Medical Ethics and Health Law, Leiden University Medical Center, Leiden University, Leiden, The Netherlands; 2 Julius Center for Health Sciences and Primary Care, University Medical Center Utrecht, Utrecht University, Utrecht, The Netherlands; 3 Department of Clinical Epidemiology, Leiden University Medical Center, Leiden University, Leiden, The Netherlands; 4 Department of Medical Humanities, Julius Center for Health Sciences and Primary Care, University Medical Center Utrecht, Utrecht University, Utrecht, The Netherlands

**Keywords:** Impracticable, informed consent, waiver, modification, infeasible, impossible

## Abstract

**Background:**

Informed consent is one of the cornerstones of biomedical research with human subjects. Research ethics committees may allow for a modification or a waiver of consent when the research has social value, involves minimal risk, and if consent is impractical to obtain. While the conditions of social value and minimal risk have received ample attention in research ethics literature, the impractical condition remains unclear. There seem to be different interpretations of the meaning of impractical within academic literature. To address this lack of clarity, we performed a systematic review on the interpretation of impractical.

**Methods:**

First, we examined international research ethics guidelines on their usage and interpretation of impractical. Next, we used international ethical guidelines to identify synonyms of the term “impractical.” Accordingly, PubMed, Embase, and Web of Science were searched for articles that included “informed consent” and “impractical” or one of its synonyms.

**Results:**

We found that there were only a few international ethics guidelines that described what could be considered impractical. Out of 2329 identified academic articles, 42 were included. Impractical was used to describe four different conditions: (1) obtaining informed consent becomes too demanding for researchers, (2) obtaining informed consent leads to invalid study outcomes, (3) obtaining informed consent harms the participant, and (4) obtaining informed consent is meaningless for the participant.

**Conclusion:**

There are conditions that render conventional informed consent truly impractical, such as untraceable participants or harm for participants. At the same time, researchers have a moral responsibility to design an infrastructure in which consent can be obtained, even if they face hardship in obtaining consent. In addition, researchers should seek to minimize harm inflicted upon participants when harm may occur as a result of the consent procedure. Invalidity of research due to consent issues should not be regarded as impractical but as a condition that limits the social value of research. Further research is essential for when a waiver of informed consent based on impractical is also reasonable.

## Background

In general, informed consent can be modified or waived after assessment by a research ethics committee. To grant such a modification or waiver, various ethical guidelines have been developed that describe conditions that must be met the following: research must have social value or serve a public good; it must involve minimal risk; and informed consent must be impractical to obtain for researchers.^1,2^ The conditions of social value and minimal risk have received ample attention in research ethics over the past years.^3–8^ The condition of impracticality, however, has remained under-investigated, despite being currently in use as a reason researchers may ask for a modification or waiver of conventional informed consent. Mosis et al.,^9^ for instance, reported that when they conducted a randomized database study to investigate the gastrointestinal tolerability of celecoxib and diclofenac in patients diagnosed with osteoarthritis who required a non-steroidal anti-inflammatory drug, general practitioners struggled to include enough patients in the study due to the informed consent procedure, even though the researchers had made ample attempts to make this as easy as possible for the practitioners. As a result of the informed consent procedure, the randomized database study became impractical to carry out, according to the researchers.^9^ There were simply not enough participants included to find meaningful outcomes.^9^ Another example of an informed consent procedure that rendered the research impractical, according to the researchers, was described by Groenhof et al.^10^ Upon evaluating their cohort study, Groenhof et al.^10^ discovered that elderly women and severely ill patients tended not to respond to the request for the use of their data for research purposes, resulting in selection bias. In emergency research, impractical is used as a condition to modify or waive conventional informed consent because there is often too little time to conduct a time-sensitive procedure and obtain informed consent from a severely ill patient.^11^ Smischney et al.^11^ described that when they compared the effects of ketofol, an alternative induction agent for severely ill patients, informed consent was waived for patients since the emergent nature of the intervention made it impractical.

In the studies that we described, informed consent apparently became impractical due to various factors, such as selection bias as a result of limited participant inclusion and limited time to obtain informed consent. Since there seem to be different conditions that make informed consent impractical, it is currently unclear when a research ethics committee should allow for a modification or waiver based on impractical. Further clarification of the meaning of impractical is needed to adequately assess if studies can be granted a waiver or modification. To clarify the meaning of impractical, we conducted a systematic review of ethical and medical literature on the interpretation(s) of impractical.

## Methods

This systematic review is reported according to the Preferred Reporting Items for Systematic Reviews and Meta-Analyses (PRISMA) statement where applicable for an ethics literature review.^12^ This systematic review was not previously registered. To perform this review, authoritative (international) ethical guidelines were first examined to find the terms that describe what “impractical to obtain conventional informed consent” means. Next, we examined how these guidelines use the term impractical and what conditions are considered to make research impractical. Sources included as follows: The Belmont Report (1979), the Council of Europe’s Convention on Human Rights and Biomedicine (1997), UNESCO’s Universal Declaration on Bioethics and Human Rights (2005), the Nuremberg Code (2007), the World Medical Association’s Declaration of Helsinki (2013), the International Ethical Guidelines for Health-related Research Involving Humans of the Council for International Organizations of Medical Sciences (CIOMS; 2016), the Clinical Trials Regulation EU No. 536/2014, the European Medicine Agency’s International Council for Harmonisation E6 (R2) Good Clinical Practice guidelines (2016), the European Union’s General Data Protection Regulation (GDPR; 2016), and the US Common Rule (2018). The terms used in these guidelines to describe impractical consisted of “infeasible,” “impracticable,” “not practical,” and “impossible.”

### Search strategy

**Figure 1.**
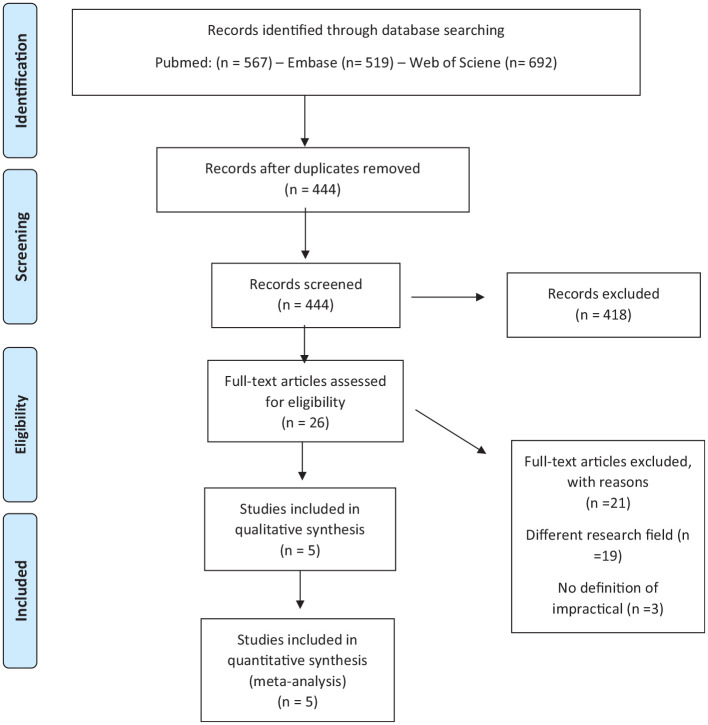
Initial search. Search string PubMed: ((“informed consent”) AND (impracticable OR infeasible OR impossible OR “not possible” OR “not feasible” OR “not practical”)). Search string Embase: ((exp Informed Consent/or informed consent.ti.) and (impracticable or impractic* or infeasible or infeasib* or unfeasible or unfeasib* or impossible or impossib* or possible or feasible or practical).ti.) not (conference review or conference abstract).pt. Search string Web of Science: ((ALL = ((“Informed Consent” OR “informed consent” OR “consent*”) AND (“impracticable” OR “impractic*” OR “infeasible” OR “infeasib*” OR “unfeasible” OR “unfeasib*” OR “impossible” OR “impossib*” OR “not possible” OR “not feasible” OR “not practical”)))) AND LANGUAGE: (English) AND DOCUMENT TYPES: (Article) Indexes = SCI-EXPANDED, SSCI, A&HCI, ESCI Timespan = 1945–2020.

After identification of the terms and the usage of impractical in the different guidelines, broad literature searches on impractical were performed. First, a PubMed/MEDLINE, Embase, and Web of Science (24 March 2020) search was conducted to identify relevant studies. We used a search strategy including the following range of keywords: “informed consent,” or “consent” in combination with “impractical,” “not practical,” “infeasible,” “not feasible,” “impossible,” and “not possible.” The detailed search strategy is presented in Figure 1. Second, we reviewed the reference sections of all articles of interest to find additional reports.

**Figure 2.**
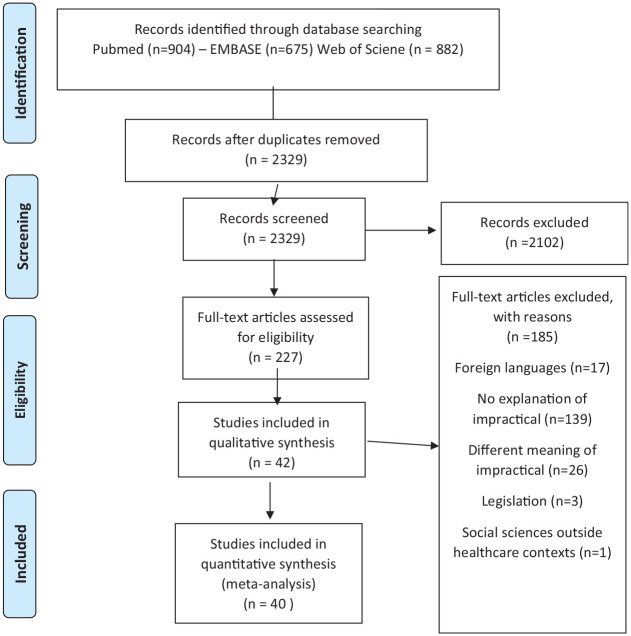
Second search. Search string PubMed: ((“Informed Consent”[Mesh] OR “informed consent”[tw] OR “consent*”[ti]) AND (“impracticable”[tw] OR “impractic*”[tw] OR “infeasible”[tw] OR “infeasib*”[tw] OR “unfeasible”[tw] OR “unfeasib*”[tw] OR “impossible”[tw] OR “impossib*”[tw] OR “not possible”[tw] OR “not feasible”[tw] OR “not practical”[tw] OR “difficult circumstances”[tw] OR “difficult circumstanc*”[tw] OR “large number”[tw] OR “large number of participants”[tw] OR “violating privacy” [tw] OR “generalizability”[tw] OR “low response rate”[tw] OR “high costs”[tw] OR “time pressure”[tw] OR “time pressur*”[tw] OR “scientific value”[tw] OR “difficult situation*”[tw])). Search string EMBASE: ((exp Informed Consent/ or informed consent.ti.) and (impracticable.ti. or impractic*.ti. or infeasible.ti. or infeasib*.ti. or unfeasible.ti. or unfeasib*.ti. or impossible.ti. or impossib*.ti. or possible.ti. or feasible.ti. or practical.ti. or difficult circumstances.ti. or difficult circumstanc*.ti. or large number.ti. or large number of participants.ti. or time pressure.ti. or time pressur*.ti. or difficult situation*.ti. or violating privacy.ti. or generalizability.ti. or low response rate.ti. or high costs.ti. or time pressure.ti. or time pressur*.ti. OR scientific value.ti. or difficult situation*.ti.)) NOT (conference review or conference abstract).pt. Search string Web of Science: (ALL = ((“Informed Consent” OR “informed consent” OR “consent*”) AND (“impracticable” OR “impractic*” OR “infeasible” OR “infeasib*” OR “unfeasible” OR “unfeasib*” OR “impossible” OR “impossib*” OR “not possible” OR “not feasible” OR “not practical”))).

A first initial search was confined to the use of the meaning of impractical in electronic health record research because we expected the use of this term to be of particular relevance for data intensive research. However, we were able to identify only one ethical analysis by Chen et al.^13^ who provide insights on possible meanings of impracticable, and four manuscripts on impractical in electronic health record research written by Angela Ballantyne’s research group.^14–17^ Due to this limited selection of articles in the field of electronic health record research, and to enrich our findings with broader insights, we decided to look for impractical and its synonyms within other research fields. These fields consisted of biobank research, emergency research, and randomized controlled trials. To broaden our search, search terms identified in key articles were added to the search string. These terms consisted of “large number of participants,” “difficult circumstances,” “time pressure,” “violating privacy,” “generalizability,” “low response rate,” “high costs,” and “scientific value.” Terms had to appear in combination with “consent.” This new search was performed on 13 April 2020. An overview of this second search strategy and key articles used can be found in Figure 2.

### Inclusion and exclusion criteria

For an article to be included in the present review, a description or interpretation of impractical or related terms in reference to obtaining conventional informed consent had to be provided by the authors. Articles written in languages other than English were excluded.

The following data were extracted from each article: author, year of publication, the context of the research, type of informed consent, and a description of the condition of impractical.

### Study selection

One of the researchers (S.J.M.L.) independently reviewed all studies by title and abstract. A second reviewer (R.v.d.G.) independently checked a random sample (20%) of the initial search outcomes, also looking at title and abstract. After the titles and abstracts were screened, one researcher (S.J.M.L.) continued reviewing the full texts and extracted the data. When in doubt, the researcher consulted the other team members (R.v.d.G. and E.S.).

## Results

### Search results

First, we examined the international guidelines on impractical. Results of this search can be found in Table 1 and in the next section. Next, we examined the international academic literature on impractical. After removing duplicates, the search yielded 2329 articles. After title and abstract screening, we screened the full text of 227 articles. Eventually, 42 articles were included (see Figure 2). In Table 2, we summarize the identified articles. Findings are reported in four categories as follows: (1) obtaining informed consent becomes too demanding on researchers, (2) obtaining informed consent leads to invalid study outcomes, (3) obtaining informed consent harms the participant, and (4) obtaining informed consent is impossible for the participant. Within these categories, articles are grouped by research category (data research, biobank research, emergency research, and randomized controlled trials research) since impractical can have different interpretations depending on the research context. An overview of the categories can be found in Table 3. The interpretations of the meaning of impractical found in the included articles do not reflect the authors’ opinions on the meaning of the term; rather they are a representation of interpretations that can be found in academic articles.

**Table 1.** Usage of impractical in research ethics guidelines.

Guideline	Description of impractical related to a waiver or a modification of informed consent
Council for International Organizations of Medical Sciences (CIOMS)—2016 International Ethical Guidelines for Health-related Research Involving Humans	“A research ethics committee may approve a modification or waiver of informed consent to research if: the research would not be feasible or practicable to carry out without the waiver or modification; the research has important social value; and the research poses no more than minimal risks to participants.”
The Declaration of Helsinki (2013)	“32. For medical research using identifiable human material or data, such as research on material or data contained in biobanks or similar repositories, physicians must seek informed consent for its collection, storage and/or reuse. There may be exceptional situations where consent would be impossible or impractical to obtain for such research. In such situations the research may be done only after consideration and approval of a research ethics committee.”
Belmont Report (2013)	–
Council of Europe’s Convention on Human Rights and Biomedicine (1997)	–
UNESCO’s Universal Declaration on Bioethics and Human Rights (2005)	“2. Scientific research should only be carried out with the prior, free, express and informed consent of the person concerned. The information should be adequate, provided in a comprehensible form and should include modalities for withdrawal of consent. Consent may be withdrawn by the person concerned at any time and for any reason without any disadvantage or prejudice. Exceptions to this principle should be made only in accordance with ethical and legal standards adopted by States, consistent with the principles and provisions set out in this Declaration, in particular in Article 27, and international human rights law.” “27. If the application of the principles of this Declaration is to be limited, it should be by law, including laws in the interests of public safety, for the investigation, detection and prosecution of criminal offences, for the protection of public health or for the protection of the rights and freedoms of others. Any such law needs to be consistent with international human rights law.”
Nuremberg Code (2007)	–
European Union’s General Data Protection Regulation (2016)	“Article 14 Information to be provided where personal data have not been obtained from the data subject 1. Where personal data have not been obtained from the data subject, the controller shall provide the data subject with the following information […]5. Paragraphs 1 to 4 shall not apply where and insofar as: (a) the data subject already has the information; (b) the provision of such information proves impossible or would involve a disproportionate effort, in particular for processing for archiving purposes in the public interest, scientific or historical research purposes or statistical purposes, subject to the conditions and safeguards referred to in Article 89(1) or in so far as the obligation referred to in paragraph 1 of this Article is likely to render impossible or seriously impair the achievement of the objectives of that processing. In such cases the controller shall take appropriate measures to protect the data subject’s rights and freedoms and legitimate interests, including making the information publicly available; (c) obtaining or disclosure is expressly laid down by Union or Member State law to which the controller is subject and which provides appropriate measures to protect the data subject’s legitimate interests; or (d) where the personal data must remain confidential subject to an obligation of professional secrecy regulated by Union or Member State law, including a statutory obligation of secrecy.”
United States Common Rule (2018)	“(3) Requirements for waiver and alteration. In order for an IRB to waive or alter consent as described in this subsection, the IRB must find and document that: (i) The research or demonstration project is to be conducted by or subject to the approval of state or local government officials and is designed to study, evaluate, or otherwise examine: (A) Public benefit or service programs; (B) Procedures for obtaining benefits or services under those programs; (C) Possible changes in or alternatives to those programs or procedures; or (D) Possible changes in methods or levels of payment for benefits or services under those programs; and (ii) The research could not practicably be carried out without the waiver or alteration.”

**Table 2.** Overview of the articles.

References	Country	Paper type	Scope of paper	Aim of paper	Terms used
1. Ballantyne	New Zealand	Opinion	Research ethics, health data	To show that a research ethics approach leads to a primary focus on individual patient consent and control of data and to show how a public health ethics lens shifts the focus to collective interests and provides for richer debate about power, justice and equity in health data research.	Impractical/impossible
2. Ballantyneand Moore	New Zealand	Opinion	Research ethics,health data	To assess how New Zealand research ethics committees weigh the potentially competing goals of enabling research and protecting patients’ rights.	Impractical/impossible
3. Ballantyneand Schaefer	New Zealand	Opinion	Researchethics, health data	To argue that a moral duty to participate in research can ground waivers of informed consent for secondary research using public sector health data, even when obtaining such consent would be practicable.	Impractical/impossible
4. Ballantyneand Schaefer	New Zealand	Opinion	Researchethics, health data	To argue that “public interest” best reflects the normative work required to justify consent waivers.	Impractical
5. Ballantyne et al.	New Zealand	Opinion	Research ethics, health data	To demonstrate the different ways researchers and research ethics committees analyze ethical questions about tissue and data research. This study is important because it demonstrates where researchers and RECs are talking past each other.	Impractical
6. Bateman et al.	The Unites States	Opinion	Stroke research	To bring the current regulations to the attention of stroke researchers and to explore their application to the conduct of acute stroke research.	Impossible
7. Bathe andMcGuire	Canada	Commentary	Research ethics,genome research	To define an ethical framework for accessing archival tissues, taking into account the needs of the research community, as well as the rights and expectations of participants.	Impractical
8. Biros et al.	The Unites States	Consensusstatement	Emergency research	To present consensus recommendation for regulatory changes for consent in emergency research.	Impractical/impossible
9. Chen et al.	The Unites States	Case study	Genetics	To explore whether a waiver of consent with notification and the option to withdraw (WNOW) is a viable alternative to written informed consent for secondary uses of samples and data.	Impractical
10. Clifton et al.	The Unites States	Researchreport	Acute brain injury	To determine the effect of consent mechanism on the conduct of one study of emergency therapy in severe brain injury, the investigators compared the data from the 392 patients who were enrolled in NABISH showing (1) the relative accrual rates, (2) the time to experimental treatment, and (3) the rates of enrollment of minorities, non-minorities, and the poor in the time period when randomization was conducted only with prospective, informed consent and in the period when waiver of consent was used in conjunction with prospective, informed consent.	Not practical
11. Colledge and Elger	Switzerland	Analysis	Bio-preservationand biobanking	To examine the implications of paragraph 25 of the Declaration of Helsinki and assess its role in the debates on proper sample handling.	Impossible/impractical/threat to validity
12. Dickert and Kass	The Unites States	Opinion	Emergency research	To deepen knowledge of how a significant and previously unstudied population—survivors of sudden cardiac death—view research in emergency settings without informed consent.	Impossibleimpractical
13. Di Iorio et al.	Italy	Opinion	Data research	To give an in-depth review of the Draft Report of the European Parliament Committee on Civil Liberties, Justice and Home Affairs and their implications for health research and statistics, should they stand as written.	Impossible/impractical
14. Drepper	Germany	Opinion	Data ethics	To argue that The European General Data Protection Regulation (GDPR) incorporates many of the principles of data protection that were already in force in the past.	Impractical/impossible
15. Flory et al.	The Unites States	Review	Informed consent	To conduct a narrative review with the goal of providing evidence and conceptual frameworks for future research and policy regarding randomization without consent.	Infeasible/impossible
16. Fox et al.	The Unites States	Research report	Trauma acute care surgeon	To examine the rationale and tradeoffs of using waiver of consent in the PRospective Observational Multicenter Major Trauma Transfusion (PROMMTT) study.	Not possible
17. Gallo et al.	Canada	Opinion	Trials	To outline the development of the use of gatekeepers in the CRT literature. To document the wide variety of roles served by gatekeepers in the protection of individual, cluster, and organizational interests in CRTs. To provide a detailed ethical analysis of the authority of gatekeepers to fulfill these roles legitimately. Fourth, and finally, we consider the application of our findings using three examples.	Impossible/infeasible
18. Gelinas et al.	The Unites States	Opinion	Research ethics	To determine when and why clinical research without consent is permissible.	Impractical/unfeasible
19. George et al.	The United Kingdom	Research report	Consent in labor research	To obtain the views of parturients on the appropriateness, need, and timing for consent for such intrapartum research, so that their views could inform the debate.	Impossible/impractical/unreasonable
20. Giraudeau et al.	France	Review	Cluster randomized trials	To assess whether participant’s informed consent was required, the nature of the consent, and whether partial information had been delivered to included participants to help prevent bias.	Impossible
21. Halila	Finland	Opinion	Emergency research	To assess different ethical arguments concerning informed consent in emergency research.	Impossible
22. London et al.	Canada	Opinion	Ethics; stepped-wedge trials	To consider whether and when ethical review is required for stepped-wedge trials.	Infeasible
23. Holman	Australia	Commentary	Methods and ethics	To argue for the impracticability to seek informed consent for use of linked health records.	Impractical
24. Jansen et al.	The Netherlands	Research report	Legal and ethical issues in intensive care medicine	To compare the results of a recent randomized controlled multicentre study in the field of intensive care medicine.	Impossible
25. Kalkman et al.	The Netherlands	Opinion	Pragmatic trials	To argue that there are salient differences between pragmatic trials, especially between premarket EPTs and pragmatic trials with standard-of-care treatments, and delineate their implications for the obligation to seek informed consent from trial participants.	Impractical
26. Kalkman et al.	The Netherlands	Opinion	Pragmatic trials	To analyze claims about the challenges that traditional informed consent procedures might pose to the practicability of pragmatic trials. Subsequently, the article discusses four alternative informed consent procedures proposed in response to these claims.	Impractical/impossible
27. Kirchhoffer and Dierickx	Australia	Opinion	Biobanking	To address the role that the concept of human dignity might play in ethical and legal reflections on the notion of informed consent in research biobanking.	Impossible
28. Lertsithichai	Thailand	Opinion	Observational research	To review the requirements for waivers of informed consent in clinical research on participants who are patients in a hospital.	Impractical
29. Lignou	The United Kingdom	Opinion	Research ethics	To discuss different types of health research employed by the cluster design where informed consent is problematic.	Infeasible/impossible
30. London et al.	The United Kingdom/Canada	Opinion	Ethics; cluster—cluster trials	To provide guidance for researchers and research ethics committees for avoiding the improper use of waivers of consent in individual-cluster trials.	Impractical/infeasible/impossible
31. Maitland et al.(2011)	The United Kingdomand Kenya	Review	Consent in emergencyresearch trials	To consider two important practical and ethical aspects of the consent process: community consultation, and alternatives to prior full written consent among populations with diminished autonomy.	Impossible/impractical
32. McGuire and Beskow	The Unites States	Opinion	Genetic research ethics	To discuss the challenge of informed consent in genetic research, explore specific elements of informed consent for genetic and genomic research, and consider alternative consent models that have been proposed.	Impossible
33. McRae et al.	Canada	Opinion	Ethical issues in cluster randomized trials	To answer the question of from whom, when, and how must informed consent be obtained in CRTs in health research?	Impractical/impossible/infeasible
34. Mentzelopoulos et al.	Greece	Review article	European Union Legislation	To review the changes in EU legislation and their impact on EU emergency research.	Impossible
35. Morgans	Australia	Opinion	Emergency health research	To explore the issues of informed consent for research in emergency health situations.	Impractical/not feasible
36. Morris	The Unites States	Ethical Analysis	Informed consent	To argue that certain exceptions to informed consent provide an ethical means to advance the science of resuscitation.	Infeasible
37. Schmit et al.	The Unites States	Delphi study	Internet research	To improve communication with patients and transparency about how complex software, such as MiNDFIRL, is used to enhance privacy in secondary database studies to maintain the public’s trust in researchers.	
38. Takala	Finland	Opinion	Medical law	To argue that there are two reasons why we should be very cautious about relaxing some of the key ethical rules such as “consent” and “confidentiality” simply to facilitate more efficient genetic research.	Impractical
39. Tu et al.	Canada	Opinion	Electronic health record research	To examine the effectiveness of the attempt to obtain consent for participation in the registry during its first 2 years of operation and describe the challenges and limitations that arose as a result.	Impractical
40. Van der Baan et al.	The Netherlands	Research report	Psychiatric biobanks	To contribute to the future organization of psychiatric hospitals, in which samples are stored and research is enabled in an ethically justifiable way.	Impractical
41. Wallis et al.	New Zealand/Ireland	Review	Electronic health record research	To investigate if research using personal health information without consent risks damaging the doctor–patient relationship.	Not feasible
42. Watanabe et al.	Japan	Opinion	Biobanking	To introduce methods used to communicate with participants in the “Biobank Japan Project (BBJP)”, which is a disease-focused biobanking project.	Impossible

CRT = cluster randomized trial; EPTs= be early pragmatic trials; RECs= research ethics committee; NABISH= National Acute Brain Injury Study: Hypothermia.

**Table 3.** Usage of impractical.

Categories	Types of research	Interpretations of impractical
1. Obtaining informed consentbecomes too demanding on researchers	Electronic health record research Biobank research Pragmatic trials Cluster randomization trials	Large sample size Investment of huge amounts of times and(monetary) resources Patient identifiers or other crucialinformation is missing Patient cannot be retraced
2. Obtaining informed consent leads to invalid study outcomes	Electronic health record research Emergency care research	Selection bias Generalizability of study results becomes compromised Participants will act differently if they knowthe study’s objective Limitation of the sample size
3. Obtaining informed consent harms the participant	Biobank research Research on pregnant women	Violation of privacy Recontacting causes social and emotional harm Informing causes undue stress for participants Prolonging painful medical procedures
4. Obtaining informed consent ismeaningless for the participant(informed consent has no value for the participant)	Biobank research Cluster randomization trials	Future research objectives are unknown Intervention is implemented on a population level No (meaningful) communication possible Lack of time Representatives cannot be identified

### Usage of impractical in international research ethics guidelines

Guidelines were found to provide definitions of impractical (see Table 1). For instance, the Council for International Organizations of Medical Sciences (CIOMS) describes that “a research ethics committee may approve a modification or waiver of informed consent to research if the research would not be feasible or practicable to carry out without the waiver or modification.”^18^ At the same time, the CIOMS guidelines do not explain when the research would not be feasible or practicable to carry out without the waiver or modification. The United States’ Common Rule also describes that informed consent may be waived or altered when “[t]he research could not practicably be carried out without the waiver or alteration.”^19^ This guidance document does not provide the reader with an interpretation of impractical, however.

### Obtaining informed consent becomes too demanding for researchers

Sixteen articles reported that conventional informed consent can become impractical when asking for it causes undue hardship for the researcher. Undue hardship for researchers was described in the context of electronic health record research when researchers had to overcome unreasonable obstacles to obtain conventional informed consent, for example, when there is a large sample size.^14–16,20–23^ In this case, researchers would have to invest huge amounts of time and (monetary) resources to obtain conventional informed consent, resulting in unworkable procedures. No papers gave precise details regarding what sample size would be considered too large, or the balance between sample size and resources that would make it possible to obtain consent. In addition, some articles reported that informed consent can become impractical when reusing old data for research purposes in electronic health record if patient identifiers or other crucial information is missing.^24–26^ Such information is often missing when the data were previously collected for other uses, such as healthcare delivery, and have been anonymized or transcribed.^27^ In addition, patients might have been relocated, passed away, or be difficult to trace.^26,28^ In biobank research, the same interpretation of impractical was provided as follows: conventional informed consent becomes impractical when participants have moved or passed away, because they are incapacitated, or because the researcher has no current contact information.^13,23,29–31^ In addition, impractical in pragmatic trials might also mean that researchers have to spend too many resources on obtaining informed consent if the conventional procedure is followed.^32^ Next, asking conventional informed consent from clusters of participants was described in several instances as impractical because of the large sample size of the study.^21,33,34^ Again, articles did not define which samples could be seen as large. One study stated that in electronic health record research, ethics committees preferred “an absolute interpretation of quantity” while “researchers favored a relative interpretation” of impractical related to “project resources available.”^22^

### Obtaining informed consent leads to invalid study outcomes

Another interpretation of impractical in the articles on electronic health record research was based on bias.^26,28^ When specific groups of eligible patients do not provide their consent, selection bias may occur and the generalizability of study results may become compromised.^14–17,25,26,28^ Two articles on the topic of pragmatic trials reported that impractical can mean that informing the participant will jeopardize the study’s outcomes because participants will act differently if they know the study’s objective.^32,35^ Furthermore, one article described that conventional informed consent might be waived in emergency research because it was impractical, in cases when only enrolling patients who were able to give their informed consent would limit the sample size, resulting in a low response rate and selection bias.^36^ In biobank research, one article also stated that in order for the informed consent procedure to be impractical, the missing data of the participants who could not be recontacted had to reduce the potential value of the data set.^13^

### Obtaining informed consent harms the participant

In biobank research, obtaining conventional informed consent could become impractical when participants might be harmed when recontacted, since they may feel that their privacy has been violated, or if contacting them can cause social and emotional harm.^29–31^ One article described the impracticability of asking conventional informed consent for research on women in labor.^37^ Obtaining conventional informed consent in this specific type of research becomes impractical when it would mean requesting it from a large number of women, of whom only a small number would actually be eligible for recruitment due to experiencing specific, worrisome circumstances when giving birth.^37^ Seeking consent from a large group of women could cause needless stress and anxiety.^37^ In addition, asking conventional informed consent for research from women in labor can sometimes be impractical since the women who suffer from these specific, worrisome circumstances have to undergo painful or harmful procedures; asking consent would mean prolonging these procedures.^37^

### Obtaining informed consent is meaningless for the participant

In biobank research, conventional informed consent was described in various articles as impractical to obtain since participants would never be truly informed this context; the use of biomaterial will only later be known to researchers.^38–42^ Rapid technological advancements make it impractical to inform a participant on the future usage of biomaterial and/or data. Both the researcher and the participant are unable to foresee all of the future research implications and risks associated with research using biospecimens, rendering informed consent meaningless since it would not be truly informed.^38^

In cluster randomized trials, obtaining conventional informed consent becomes impractical since the intervention is implemented on a population level; participants are not able to provide meaningful consent since they would be unable to escape the intervention.^21,33,34,42–44^

In emergency care research, conventional informed consent was generally described as impractical to obtain since it was often not possible to have a (meaningful) conversation with the patient (or her representatives) due to stress and anxiety, or in certain cases because the patient was in a coma or suffering from serious injuries.^45–47^ In addition, the acute character of emergency care research was mentioned as one of the main obstacles for obtaining informed consent, as certain medical conditions can develop rapidly.^48,49^ Lack of time to start the (lifesaving) intervention, to start treatment, or to locate the representative for proxy consent was described as making conventional informed consent impractical to obtain.^45,50^ Representatives must legally authorize research when a patient is unable to do so. In an emergency situation, there is often insufficient time to locate such representatives within the time window in which the research can take place.^47,48,51–55^ In emergency research, informed consent is often sought after capacity is regained in order to promote respect for autonomy.

## Conclusion

When informed consent is considered impractical, it may have different meanings. For researchers, impractical can imply having to face difficulties with the inclusion of large numbers of participants, unreachability of participants, or a lack of necessary resources. The literature also mentions that “impractical” for researchers implies that informed consent may introduce bias into the study. For the participant, impractical can imply that providing informed consent would harm her, violate her privacy, or be meaningless.

As mentioned in the introduction, there are three necessary conditions that need to be met for researchers to ask for a modification or waiver for informed consent as follows: impractical, minimal risk, and social value. For impractical to have independent meaning and weight, its meaning must be distinct from that of the other two conditions. In our search, bias due to the informed consent procedure was often used as an “impractical” argument. Impractical, however, can refer to (1) the study protocol and (2) the study objective. Although obtaining informed consent can *cause* a selection bias, it might still be *practicable* to request it from participants. The study’s design is still practicable and can be carried out, even though the study’s objective—to collect reproducible, valid knowledge—can no longer be met. In other words, there are no feasible alternatives, and the study objective cannot be met at the same time as fulfilling the social value requirement. When informed consent leads to bias the question for a research ethics committee will be whether there is still sufficient social value to obtaining informed consent, and not whether it is impractical.

It remains unclear whether there is a threshold for informed consent to be regarded as impractical. In some cases, it will truly be morally impossible to ask for informed consent, for instance, when a patient has passed away or when a person cannot possibly be found since data used in the research are anonymous and untraceable. In addition, many articles describe that informed consent becomes impractical to obtain when there are a large number of research participants involved.^14–16,21^ Yet none of these studies defines exactly how many participants are considered too many to ask for informed consent.

However, even if obtaining informed consent is hampered by a having to ask a large number of participants, which may lead the research ethics to grant a waiver of informed consent, researchers could still do various things to ensure respect for the autonomy of participants. Researchers in electronic health record research, in contrast to emergency research settings, still have sufficient time to make provisions. The time argument made in emergency research contexts related to impractical in asking for informed consent is substantially different in electronic health record research.

Impractical might also be interpreted as meaningless or harmful for the participant. In cluster randomized trials, conventional informed consent might become impractical because participants are oftentimes allocated to study arms before consent for randomization is obtained, and thus may not be able to escape the intervention. In the context of electronic health record research, there is no similar situation that would imply meaninglessness of consent for the participant. However, the issue of harm to the participant can be translated to electronic health record research. In electronic health record research, harm can be interpreted as social or mental harm, which may occur when participants are reminded of unfortunate events—such as the death of a loved one or newborn—when asked to participate in research, or when they are confronted with their disease. Research ethics committees should decide if there is indeed harm inflicted when participants are asked to provide their consent for electronic health record research and whether it can be minimized.

When researchers apply for a waiver of informed consent based on the impractical condition, the research ethics committee may take our menu of potential interpretations of impractical and accordingly ask the researchers to explain whether their reasoning is sound (e.g. whether obtaining informed consent is indeed too demanding because it is too expensive). If the research ethics committee considers the researchers’ arguments to be invalid, the committee may reject the protocol or accept a protocol that has been modified at this point.

However, the literature that we reviewed does not touch upon the underlying ethical issue whether a waiver of informed consent based on impractical is also always *reasonable*. A rejection of the impractical condition will have wider implications that a research ethics committee should consider. When the research ethics committee does not grant a waiver of informed consent based on the impractical condition, the committee should also consider the implications of a protocol rejection or modification. That is, they should consider a change in study objectives that might be necessary, taking more time for the study to be completed, alternative design approaches that might be essential, resolving the research question with slightly less accuracy or precision, implications for resources to be spent on other studies, or even no conduct of the study at all. At the same time, research ethics committees that may be too permissive and (easily) allow for acceptance of impractical as a condition may run the risk that researchers design their studies in such a way that informed consent is infeasible (for instance, by cutting down on costs and logistics). In other words, this condition necessitates the research ethics committee to seek a fine balance between being too permissive and too restrictive, taking into account scientific validity and social value, the scarcity of resources, and even the credibility of the research ethics committee itself when it becomes known for easily accepting the argumentation for this condition. Therefore, further ethical analysis of this concept, and especially the position of this concept in the complex decision-making structure of a research ethics committee, remains essential.

This review has some limitations that should be taken into account when interpreting the results. One such limitation is the focus on international guidelines and the exclusion of non-English research papers. A closer view of national guidelines and papers might have provided a more thorough interpretation of impractical, and it may be that they have a more elaborate understanding of this condition. Our own Dutch guidelines, for instance, touched on impractical but did not give an elaborate interpretation of it (Federa Code of Conduct, 2019). In addition, the international ethical guidelines that we employed to find synonyms of impractical can be viewed as European/American. We did not use other international ethical guidelines. There might have been other synonyms for impractical used in publications that we did not know of.

We hope that this paper will be seen as an open invitation for international ethical guideline committees and ethicists to further clarify the interpretation of impractical. It may be interesting to do a review study of national research ethics guidelines on the different national interpretations of informed consent. In addition, reviewing the work of different research ethics committees that have allowed for waivers and modifications of consent could also be informative. By looking at their decisions, we might be able to come to a better understanding of when it is impractical to obtain consent.
